# The WRKY transcription factor PlWRKY65 enhances the resistance of *Paeonia lactiflora* (herbaceous peony) to *Alternaria tenuissima*

**DOI:** 10.1038/s41438-020-0267-7

**Published:** 2020-04-01

**Authors:** Xue Wang, Junjie Li, Jing Guo, Qian Qiao, Xianfeng Guo, Yan Ma

**Affiliations:** 1 College of Forestry, Shandong Agricultural University, No. 61, Daizong Road, 271018 Tai’an, Shandong China; 2 Shandong Provincial Research Center of Demonstration Engineering Technology for Urban and Rural Landscapes, 271018 Tai’an, Shandong China; 3 Shandong Institute of Pomology, 271000 Tai’an, Shandong China

**Keywords:** Plant stress responses, RNAi, Plant stress responses, RNAi

## Abstract

In this study, the disease resistance gene *PlWRKY65* was isolated from the leaves of *Paeonia lactiflora* and analyzed by bioinformatics methods, and the localization of the encoded protein was explored. Quantitative real-time PCR (qRT-PCR) was also used to explore the response of this gene to *Alternaria tenuissima*. The results showed that the gene sequence contained multiple cis-acting elements involved in the response to hormone signaling molecules belonging to the IIe subgroup of the WRKY family, and the encoded proteins were located in the nucleus. The *PlWRKY65* gene has a positive regulatory effect on *A. tenuissima* infection. After silencing the *PlWRKY65* gene via virus-induced gene silencing (VIGS), it was found that the gene-silenced plants were more sensitive to *A. tenuissima* infection than the wild plants, exhibiting more severe infection symptoms and different degrees of changes in the expression of the pathogenesis-related (PR) genes. In addition, we showed that the endogenous jasmonic acid (JA) content of *P. lactiflora* was increased in response to *A. tenuissima* infection, whereas the salicylic acid (SA) content decreased. After *PlWRKY65* gene silencing, the levels of the two hormones changed accordingly, indicating that *PlWRKY65*, acting as a disease resistance-related transcriptional activator, exerts a regulatory effect on JA and SA signals. This study lays the foundation for functional research on WRKY genes in *P. lactiflora* and for the discovery of candidate disease resistance genes.

## Introduction

Plant immunity is a complex, multilayered system that includes several defense mechanisms. In addition to passive defense mechanisms, such as the cuticle, cell wall, and antimicrobial compounds, plants exhibit two layers of active defense mechanisms. The first is pattern-triggered immunity (PTI), induced by pathogen-associated molecular patterns (PAMPs), and the second is effect-triggered immunity (ETI), induced by pathogen proteins^1^. When an immune response occurs in plants, the expression of disease resistance genes in cells is regulated by transcription factors (TFs), including the WRKYs, which constitute one of the largest transcription factor families and are involved in disease resistance^2^.

The WRKY TF family is named for the highly conserved WRKYGQK heptapeptide sequence in the N-terminal amino acid sequence of its members^3^. Because of the presence of different numbers of WRKY domains and different zinc finger motifs, the WRKY TFs can be divided into three groups. Based on the phylogenetic tree of WRKY protein domains, group II can be divided into five subgroups (IIa–e)^4^. Studies have shown that when induced by external stimuli in plants, WRKY TFs are regulated by a cascade of defense signaling networks that bind with the promoters of downstream genes to regulate their expression and enhance plant defense^5^. Among plant disease resistance-related responses, the most widely studied is the regulation of the salicylic acid (SA) and jasmonic acid (JA) signaling pathways. A great deal of scientific research has demonstrated that the WRKY family is directly or indirectly involved in these two signaling pathways^6^. For example, *GbWRKY1* mediates the disease resistance and development of cotton through the JA signaling pathway and the negative regulation of cotton resistance to *Botrytis cinerea*^7^. Rice *OsWRKY13* can directly or indirectly regulate the expression of upstream and downstream genes of JA and SA to inhibit JA synthesis-related and JA-responsive gene expression and activate SA synthesis-related and SA-responsive gene expression to participate in disease resistance in rice^8^.

Herbaceous peony is a common ornamental plant in China that has both economic and cultural value. Red spot is a serious disease of herbaceous peony caused by *Alternaria alternata* (*A. alternata*) and *Alternaria tenuissima* (*A. tenuissima*)^9^ that hinders peony production and causes serious economic loss. Moreover, the large-scale infection of peonies affects the growth and development of the underground bud, thereby affecting plant production in the second year. In recent years, the roles of WRKY TFs in disease resistance in plants have been widely reported. In *Solanum pimpinellifolium*, *SpWRKY1*, *SpWRKY3*, and *SpWRKY6* participate in resistance against *Phytophthora infestans* as positive regulators^10–12^. Overexpression of *OsWRKY13* may improve the ability of rice to resist bacterial blight, which is a common problem in rice^8^. However, no studies on the response of the *Paeonia lactiflora* WRKY family to pathogen stress have been reported. In this research, we identified a differentially expressed gene, *PlWRKY65*, from transcriptome data collected under infection with *A. tenuissima*. Studies have shown that *PlWRKY65* can positively regulate the resistance of herbaceous peony to *A. tenuissima* and may play a role through either direct or indirect involvement in SA- and JA-mediated disease resistance signaling pathways.

## Results

### Cloning and sequence analysis of *PlWRKY65*

**Fig. 1:**
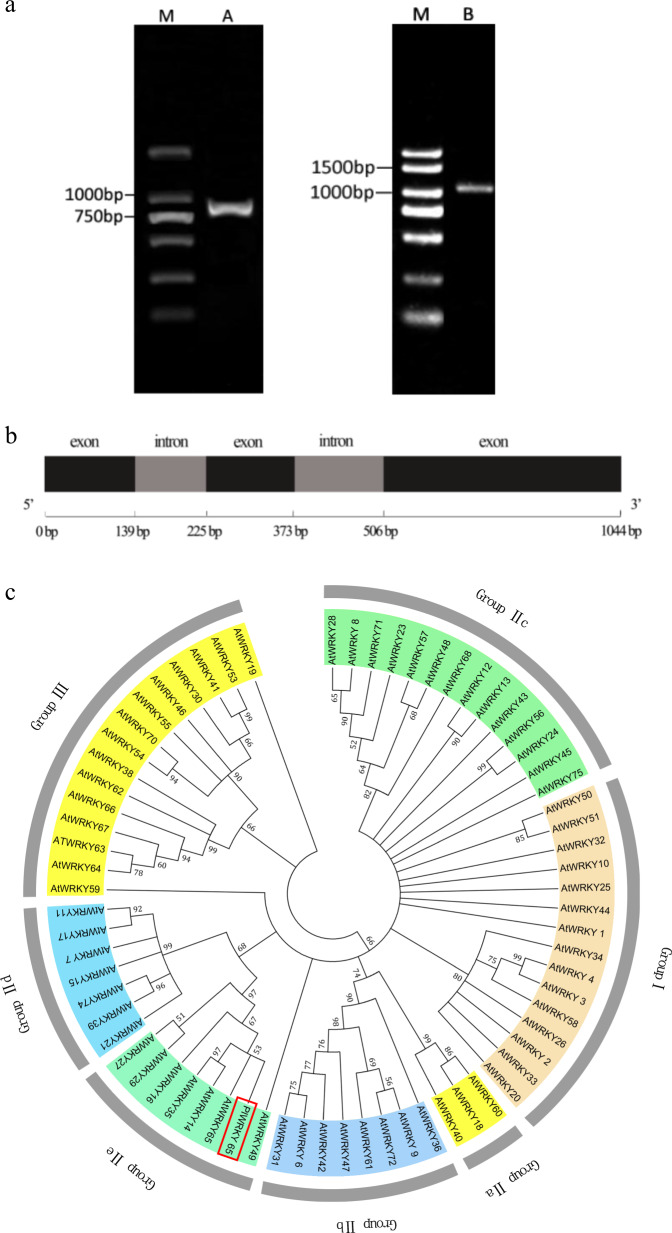

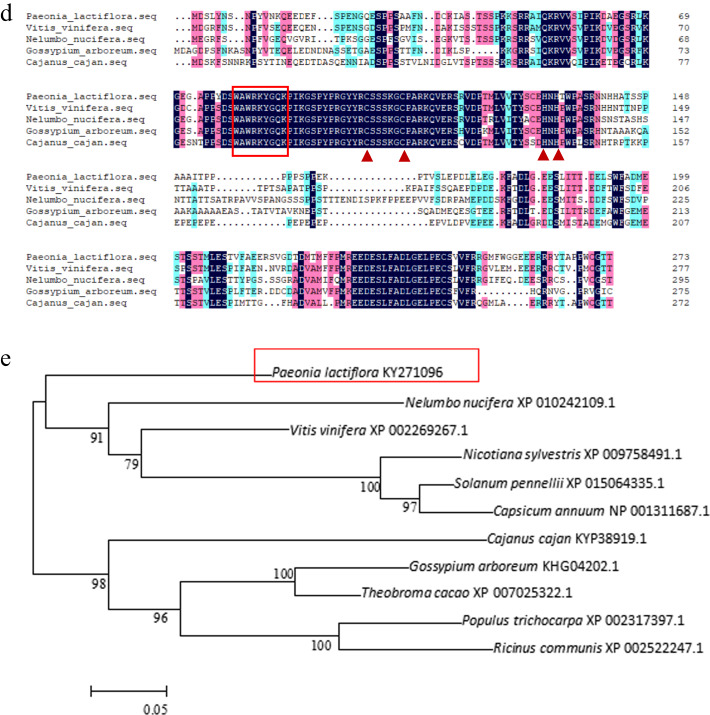
Bioinformatics analysis of *PlWRKY65*. **a** PCR amplification products of *plWRKY65*: M, DNA marker DL2000; A, *PlWRKY65* cDNA fragment; B, *PlWRKY65* genomic DNA fragment. **b** Structure of the *PlWRKY65* sequence. **c** Phylogenetic analysis based on the amino acid sequences of PlWRKY65 and WRKY family genes in *Arabidopsis*. **d** Multiple alignment of the deduced PlWRKY65 amino acid sequences with its homologs. Note: The box indicates a WRKYGQK heptapeptide sequence; the triangle indicates the zinc finger structure. **e** The phylogenetic tree derived from the alignment of the amino acid sequences of PlWRKY65 and other WRKY65

The genomic DNA and cDNA of *P. lactiflora* ‘Da Fugui’ were used as templates for amplification, and sequences with lengths of 1044 and 825 bp were obtained, respectively. The corresponding GenBank accession number is KY271096. The obtained gene was named *PlWRKY65* due to its high homology with *Arabidopsis AtWRKY65* (Fig. 1a). By comparing genomic DNA with cDNA sequences, *PlWRKY65* was found to contain three exons and two introns (Fig. 1b), and the second intron was a typical R-type intron in the conserved WRKY domain.

Furthermore, PlantCARE software was used to analyze the *PlWRKY65* gene sequence, and the results confirmed that the intron sequences of *PlWRKY65* contain 14 CAAT box components, 6 TATA box components, G-box light-responsive components, several hormone signaling molecule-responsive *cis*-elements, such as methyl jasmonic acid (MeJA) signal elements, and *cis*-elements involved in the metabolic regulation of proteins.

A phylogenetic tree of PlWRKY65 and *Arabidopsis* WRKY family genes was constructed by referring to the standard classification of *Arabidopsis* WRKY members to carry out phylogenetic analysis and clustering^3^. The results showed that PlWRKY65 and AtWRKY65 formed a branch belonging to the IIe subgroup of the WRKY family (Fig. 1c). Multiple comparative analyses indicated that the PlWRKY65 gene-coding region contains a WRKY domain structure consisting of 58 (152–209) amino acids, and this structure shares high homology with other plant WRKY65 TFs. In the N-terminal domain, the conserved structure contains the highly conserved WRKYGQK heptapeptide sequence, and the C-terminus harbors a C_2_H_2_ (CX_5_CX_23_HNH) zinc finger-type structure (Fig. 1d). The phylogenetic tree results showed that among the analyzed WRKY65 proteins, *P. lactiflora* PlWRKY65 was the most closely related to *Nelumbo nucifera* NnWRKY65 (Fig. 1e).

### Subcellular localization of the PlWRKY65 protein

**Fig. 2:**
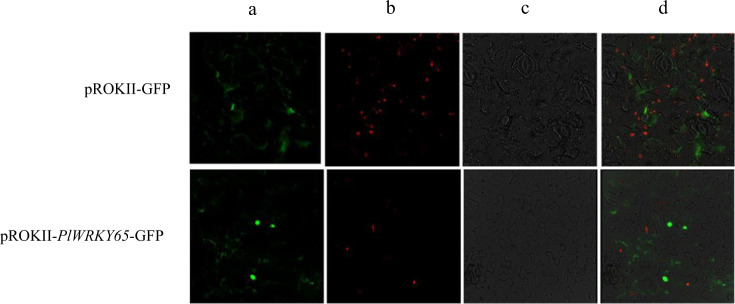
Subcellular localization of the *PlWRKY65* protein in *Nicotiana benthamiana*. **a** Fluorescent image, **b** chloroplast auto-fluorescence image, **c** natural light image, and **d** merged image

We transformed the recombinant vector pROKII-*PlWRKY65*-GFP and the vector containing only GFP into tobacco leaf epidermal cells to study the specific sites of the PlWRKY65 protein in cells. The results showed that green fluorescence in the epidermal cells of tobacco leaves transformed with the recombinant vector was present only in the nucleus, while in the leaves of the control group, the fluorescence showed a diffuse distribution and was present in both the nucleus and cytoplasm. Thus, it was inferred that the PlWRKY65 protein is present in the nucleus and may play a role there (Fig. 2).

### *PlWRKY65* was positively induced by *A. tenuissima*

**Fig. 3:**
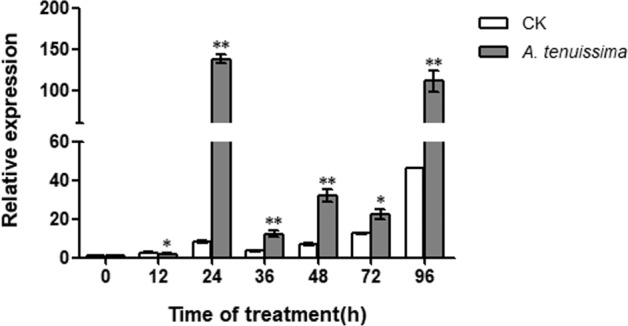
Expression patterns of *PlWRKY65* under infection with *A. tenuissima*. *indicates a significant difference between the treatment and control (0.01 < *P* < 0.05); ** indicates an extremely significant difference (*P* < 0.01); the same below

Based on the differential expression of *PlWRKY65* according to the transcriptome data, we further studied the expression patterns of *PlWRKY65* after infection with *A. tenuissima*. The results showed that *PlWRKY65* was positively expressed during infection with *A. tenuissima*. The expression of *PlWRKY65* increased sharply and peaked at 24 h after infection with *A. tenuissima*, at a level 16.76 times higher than that of the control. Thereafter, the expression level remained higher than that of the control group until 96 h, showing an overall upward trend (Fig. 3).

### VIGS of *PlWRKY65* reduced the transcript abundance of endogenous *PlWRKY65*

**Fig. 4:**
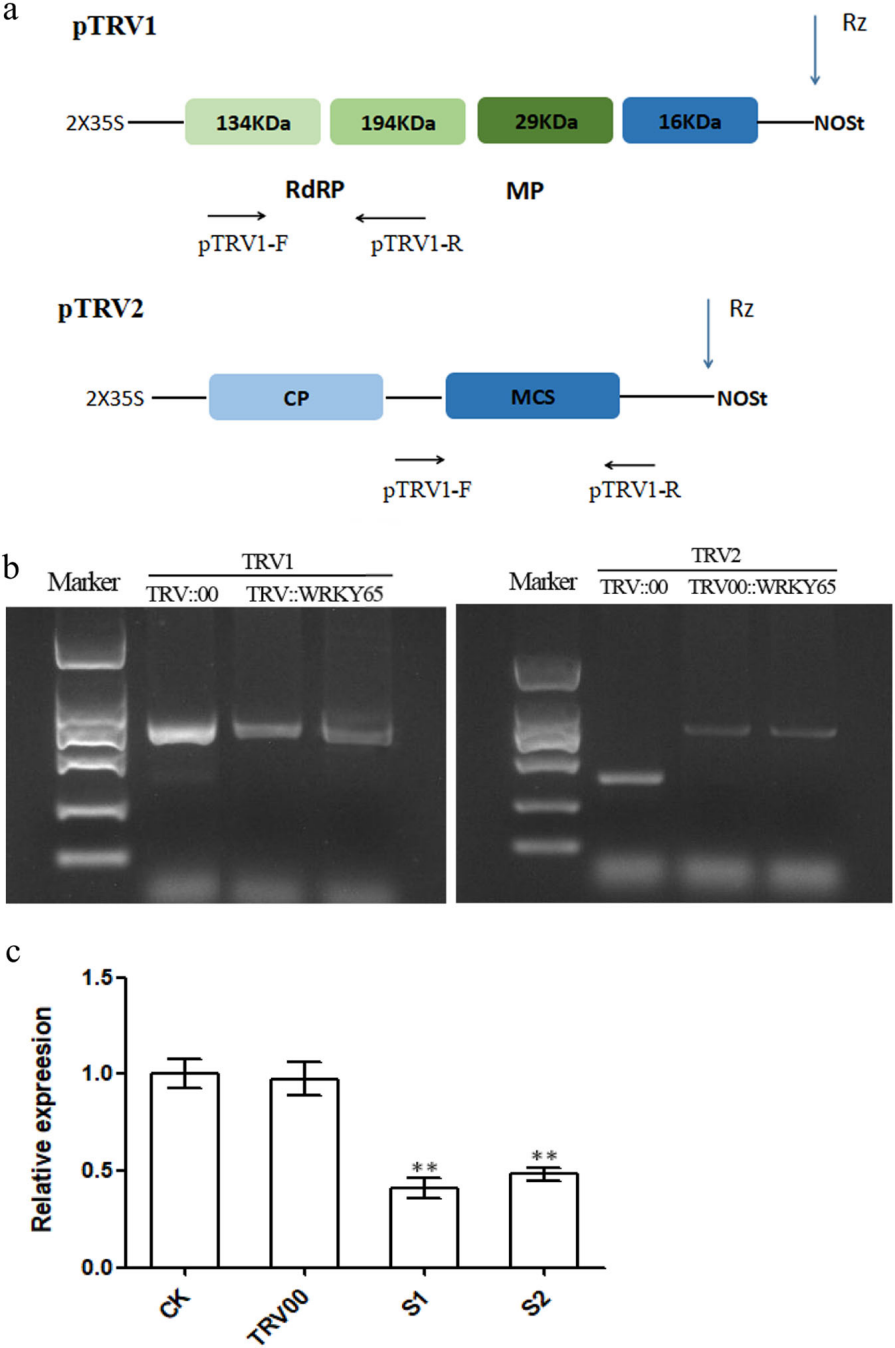
The effect of *PlWRKY65* silencing in VIGS plants. **a** Schematic diagram of the pTRV1 and pTRV2 plasmids and primers. **b** The PCR identification of RNA1 and RNA2 of TRV in *P. lactiflora* leaves. **c** *PlWRKY65* gene expression levels in *PlWRKY65*-silenced plants. S1 and S2 are both TRV::WRKY65 plants and are independent of each other

To preliminarily understand the mechanism of *PlWRKY65* in response to infection with *A. tenuissima*, we further evaluated the functions of *PlWRKY65* by silencing. Leaf cDNAs obtained after 10 days of infection with the empty vector TRV::00 and recombinant vector TRV::WRKY65 were used as templates, and pTRV1-F/R and pTRV2-F/R were used for PCR-based detection (Fig. 4a). The results showed that the target bands of 647 bp (pTRV1) and 372 bp (pTRV2) could be amplified from the *P. lactiflora* samples inoculated with TRV::00, and bands of 647 bp (pTRV1) and 774 bp (pTRV2::WRKY65) could be amplified from the samples inoculated with TRV::WRKY65 (Fig. 4b). These findings indicated that TRV::00 and TRV::WRKY65 were successfully inserted and expressed in the genome of *P. lactiflora*.

The silencing of the *PlWRKY65* gene in the leaves of *P. lactiflora* was detected by qRT-PCR. The leaves were treated with TRV::00 or TRV::WRKY65 (with blank control), and after 16 days of treatment, the leaves were collected, and RNA was extracted. The results showed that *PlWRKY65* gene expression in the leaves infected with TRV::WRKY65 was markedly lower than that in the blank control and the leaves infected with TRV::00 (Fig. 4c). These findings indicated that *PlWRKY65* was effectively silenced in TRV::WRKY65-infected leaves.

### *PlWRKY65*-silenced plants exhibited greater sensitivity to *A. tenuissima*

**Fig. 5:**
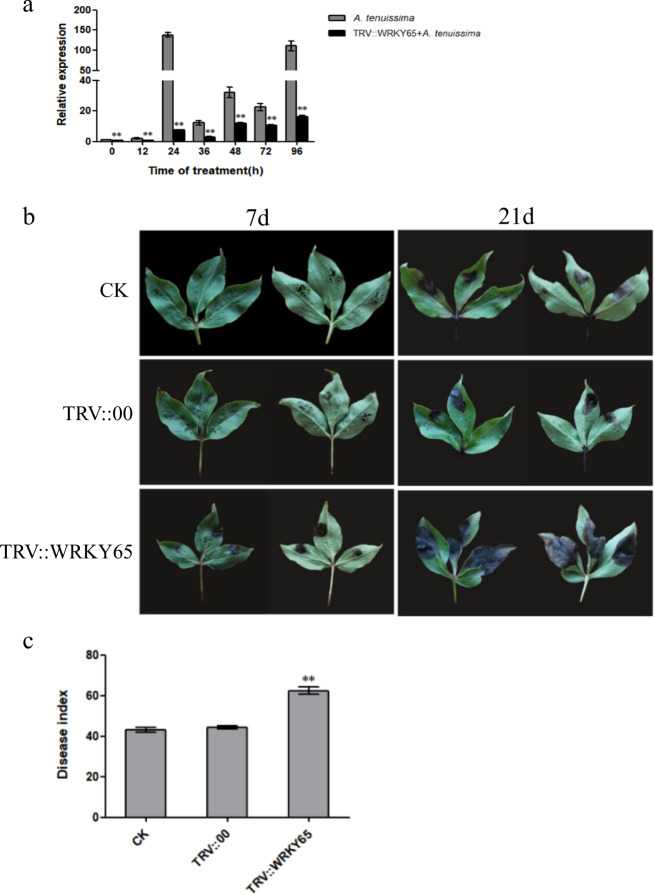
Phenotypic differences in *PlWRKY65*-silenced plants in response to *A. tenuissima*. **a** Expression of *PlWRKY65* in response to *A. tenuissima* before and after gene silencing. **b** Leaf phenotype under different treatments. **c** Disease index of plants treated with different treatments

The control plants and the plants silenced for 20 days (silencing efficiency > 60%) were selected for the pathogen infection test. As shown in Fig. 5a, the expression of *PlWRKY65* in response to infection with *A. tenuissima* in the *PlWRKY65*-silenced plants was markedly lower than that in the two control groups, but the changes in expression were consistent with those of the control plants. These findings indicated that the TRV-*PlWRKY65-*silencing vector effectively inhibited the expression of the *PlWRKY65* gene, and in the *PlWRKY65*-silenced plants, *PlWRKY65* still played an active regulatory role against *A. tenuissima*.

After infection with *A. tenuissima*, *PlWRKY65*-silenced plants exhibited higher sensitivity to the pathogens than the control plants (Fig. 5b), which was characterized by severe spreading of disease spot areas and parched perforation. After 21 days of infection, the incidence statistics of the plants showed that all plants were infected by *A. tenuissima*, and the disease indexes of the blank control plants, empty vector control plants, and *PlWRKY65-*silenced plants were 43.05, 44.10, and 62.45, respectively. The disease index of the *PlWRKY65-*silenced plants was markedly higher than that of the control (Fig. 5c) and was 19.40% and 18.35% higher than those of the blank control group and the empty vector control group, respectively. All of the above results illustrated that the *PlWRKY65* gene was involved in the resistance of peony to *A. tenuissima* and played an active role in regulating disease resistance in peony by increasing its expression.

### *PlWRKY65* expression participates in JA and SA signaling

**Fig. 6:**
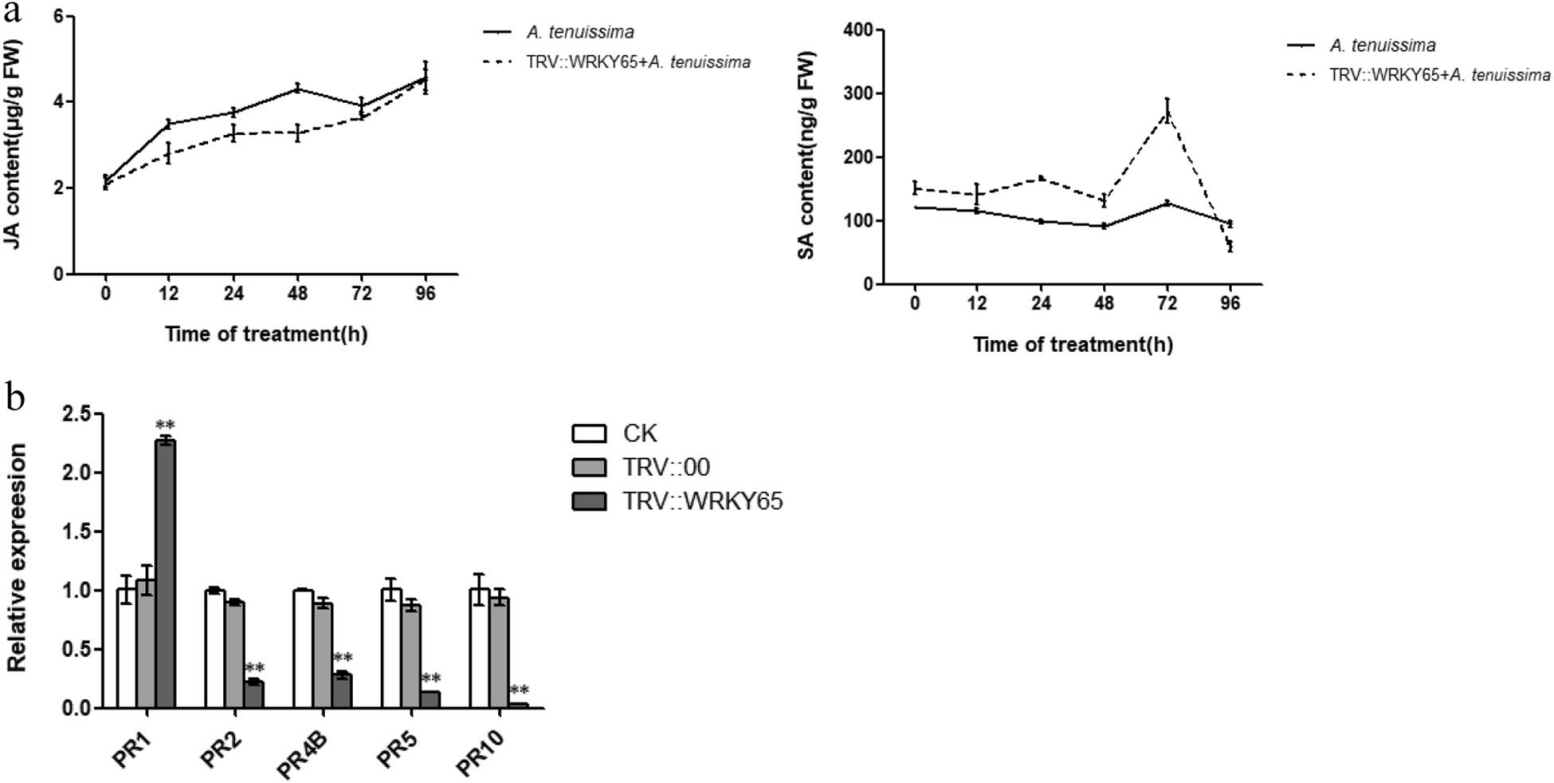
Endogenous hormone concentrations and expression of pathogenesis-related genes. **a** Endogenous hormone contents in the leaves of *P. lactiflora* treated with *A. tenuissima.* **b** Expression of pathogenesis-related genes in the control and gene-silenced leaves

To investigate whether endogenous hormones respond to resistance to *A. tenuissima* and whether *PlWRKY65* is associated with related hormones, we detected the endogenous levels of JA and SA in *PlWRKY65*-silenced plants and normal plants infected with *A. tenuissima* over 96 h. The results revealed (Fig. 6a) that the JA content of the control plants increased overall from 0 to 96 h, reaching the highest level at 96 h. However, SA decreased from 0 to 48 h, then increased to a peak at 72 h and decreased again thereafter. Interestingly, the JA level in the *PlWRKY65-*silenced group was lower than that in the normal group and gradually recovered to the same level as that in the normal plants at 96 h, but the SA level in the *PlWRKY65*-silenced group was higher than that in the normal group and recovered to the same level as that in the normal plants at 96 h, or even to a slightly lower level than that in the control. These data revealed that the changes in endogenous JA and SA levels are closely related to the disease resistance process after infection with *A. tenuissima* and are specifically correlated with the expression of *PlWRKY65*.

### Differential regulation of pathogenesis-related genes by *PlWRKY65*

We conjectured that *PlWRKY65* might be involved in the JA and SA signaling pathways because of the changes in endogenous hormones in *PlWRKY65*-silenced plants and the hormone signal-response elements in the *PlWRKY65* gene. Therefore, we used qRT-PCR to analyze the expression of the JA and SA pathogenesis-related (PR) genes PR1, PR2, PR4B, PR5, and PR10 to verify this hypothesis. The results showed that only *PlPR1* expression in *PlWRKY65*-silenced plants was markedly higher than that in control plants after 96 h of infection with *A. tenuissima*, while the expression of the other four *PlPRs* in *PlWRKY65*-silenced plants was markedly lower than that in the control plants (Fig. 6b). These results further indicated that *PlWRKY65* regulated the resistance of *P. lactiflora* to *A. tenuissima* by participating in the JA and SA signaling pathways.

## Discussion

The transcriptional regulation of defense genes plays indispensable roles in plant resistance. Therefore, the identification of the components of the plant defense system and corresponding response pathways is an important step for understanding plant stress resistance. The WRKY TF family, which is involved in disease responses, was identified recently, and many of its members are involved in the pathogen resistance response, as shown for the *Arabidopsis thaliana* WRKY7, 33, 22, 70, and 54 TFs, which are directly involved in resistance to fungi^13–16^. However, most studies on these TFs have been conducted in *A. thaliana*, tomato, tobacco, potato, rice, cotton, and other plants^17–21^, and research on the gene functions of peony WRKY TFs is very limited. We screened a differentially expressed *PlWRKY65* gene from the transcriptome data of peony, speculated that it was related to the regulation of disease resistance, and provided strong evidence of the involvement of this gene in disease response regulation.

Regulatory elements involved in gene expression have been found in the introns of many genes. The introns of *PlWRKY65* contain TATA box components, G-box light-responsive components and several hormone signaling molecule-responsive cis-elements. These characteristics indicate that the introns of this gene may increase the transcription of the gene and participate in hormone regulation or synthesis and other plant developmental processes. Theoretically, we can establish a comparison between WRKY TFs from other plants and peony WRKYs and infer the potential functions of homologous WRKY proteins according to the known functions of these proteins. Previous studies have shown that the *Arabidopsis AtWRKY65* gene is involved in the immune response related to *AtFLD*^22^. Based on the fact that *PlWRKY65* and *AtWRKY65* both belong to the IIe subgroup, it is speculated that *PlWRKY65* may be involved in the stress response during plant growth. The subcellular localization results implied that the PlWRKY65 protein was present in the nucleus, which agreed with the results obtained for WRKY proteins in other species^23–25^. Therefore, we speculated that the PlWRKY65 protein acts as a transcriptional regulator, similar to most other WRKY proteins, and activates the expression of downstream target genes by binding to the W-boxes of target genes.

TFs, especially those of the WRKY family, play an important role in plant disease resistance responses as signaling pathway regulators. *AtWRKY8* and *AtWRKY28*, which belong to the IIa subgroup, can positively regulate the resistance of plants to *B. cinerea*^26,27^. Studies on the overexpression of *OsWRKY45-1* and *OsWRKY45-2* showed that this pair of alleles had a positive regulatory effect on resistance to *Magnaporthe grisea*, a fungal pathogen of rice^6^. The same conclusion was reached in this study, in which the expression of *PlWRKY65* increased under induction by *A. tenuissima* and positively regulated the disease resistance of peony. However, in tomato, *SlWRKY70* transcription negatively regulates plant resistance to fungi^28^. Therefore, WRKY TFs may be positively or negatively regulated to participate in the disease resistance-related responses of plants. In the experiment involving *A. tenuissima* infection, all treatments were carried out under natural light, and because *A. tenuissima* infection occurred during the day, sampling at 12 and 36 h after infection was conducted at night. The experiment revealed that *PlWRKY65* expression in the blank control group was similar at 12 and 36 h but slightly higher at 24 and 48 h (Fig. 3). These results, together with the results for the G-box light-responsive components in the gene sequence, indicate that the expression of *PlWRKY65* may be induced by optical signals. At 48 h after infection, the outdoor temperature plummeted, accompanied by rain, which may have been the reason for the significant increase in expression in the blank control at 72 and 96 h.

It has been found that plants infected by pathogens tend to accumulate plant protectors, such as phytoalexins, scopolin, and scopoletin around the invasion site, and the synthesis of these protectors depends on the JA, SA, and ethylene signaling pathways in plants^29–31^. However, most studies have revealed that WRKY TFs can mediate multiple disease resistance response pathways to regulate the expression of disease resistance-related genes, including the JA and SA signaling pathways^6^. In *Catharanthus roseus*, JA can promote the transcriptional accumulation of *CrWRKY1*, *CrWRKY8*, *CrWRKY13*, and *CrWRKY38*^32^. Under MeJA treatment, *GhWRKY40* gene expression is upregulated and positively regulates the resistance of cotton plants to *Ralstonia solanacearum*^33^. Rice *OsWRKY13* is co-induced by SA and JA, and studies have surprisingly shown that *OsWRKY13* in turn affects the accumulation of SA; that is, plants that overexpress *OsWRKY13* accumulate more free SA^8^. In this study, *PlWRKY65* silencing caused a decrease in JA content and an increase in SA content, which indirectly indicated that *PlWRKY65* might promote JA accumulation and inhibit SA synthesis.

In the complex network through which plants regulate their response to external injury, there is an obvious antergic relationship between the SA and JA signaling pathways, and WRKY TFs mostly play a regulatory role at the junction of their signaling pathways^34^. Rice *OsWRKY13*, as a TF that directly or indirectly regulates disease resistance via the SA and JA signaling pathways, activates the transcription of SA synthesis-related genes and SA-induced genes and inhibits the expression of JA synthesis-related genes and JA-induced genes^8,35^. *Erwinia carotovora* infection in wild-type *Arabidopsis* can induce increased *AtWRKY70* gene expression, which has been proven to be associated with elevated levels of endogenous SA. At the initial stage of infection, the transient increase in the JA level inhibits the expression of *AtWRKY70*. Studies have concluded that *AtWRKY70* is a transcriptional activator of SA-inducing genes and a transcriptional repressor of JA-inducing genes, leading to the intersection of the SA- and JA-mediated signal defense pathways^36^. Our research clearly showed that after infection with *A. tenuissima*, the endogenous JA content increased, but the content of endogenous SA decreased, and the two hormones showed opposite trends 96 h after infection. These results not only confirmed the mutual inhibition of the two hormones but also suggested that *PlWRKY65* may be positively regulated in the JA-mediated signaling pathway and may inhibit SA signaling pathway regulation. SA is an indispensable regulator that mainly combats infections involving biotrophic pathogens, such as *Hyaloperonospora parasitica* and *Oidium neolycopersici*^37^; however, JA-related defense mechanisms protect against necrotrophic pathogens such as *Alternaria brassicicola* and *Botrytis cinerea*^38,39^. As a necrotrophic pathogen, *A. tenuissima* is likely to cause the activation of JA signaling when it infects plants, which further explains the increase in JA content after infection with *A. tenuissima*.

The sudden onset of PTI and ETI in plants is usually regulated by hormone signaling pathways. The best studied of these pathways are those involving SA, JA, and ethylene, and these endogenous hormones can induce the expression of *PR* genes^40,41^. In *Arabidopsis*, SA activates the expression of the *AtPR-1*, *AtPR-2,* and *AtPR-5* genes, while JA activates the expression of the *AtPR-3*, *AtPR-4*, and *AtPR-12* genes^42^. MeJA can induce the expression of *MaPR5-2* and *MaPR5-3*, while both SA and MeJA can induce high *MaPR1-1*, *MaPR2*, and *MaPR10c* expression in banana fruit^43^. Signal transduction in the plant immune response is dependent on TFs. It has been reported that some pathogens and pathogen-derived elicitors, including SA and JA, can induce the expression of WRKY TFs, and some WRKY TFs are in turn involved in both the SA and JA pathways, showing the intersection of these effects^5,44^. Furthermore, WRKY TFs can regulate the expression of some *PR* genes by binding to their promoters^5^. Rice *OsWRKY3* can promote the expression of *OsPR1* downstream as a transcriptional activator^35^. In rice, transgenic lines overexpressing *OsWRKY28*, *OsWRKY71*, *OsWRKY76*, and *OsWRKY62* activate the pathogenesis-related gene *OsPR10*, contributing to resistance against *Xanthomonas oryzae* pv. *oryzae* (Xoo)^45^. In our study, decreased *PlWRKY65* expression resulted in significantly decreased *PlPR2*, *PlPR4B*, *PlPR5,* and *PlPR10* expression and upregulated the transcriptional abundance of *PlPR1*. This illustrates that *PlWRKY65* participates in the regulation of these *PlPRs* and may be involved in JA-mediated and SA-mediated signaling pathways. An important finding is that *PlPR1* is likely to be highly induced by SA and participate in the SA signaling pathway.

**Fig. 7:**
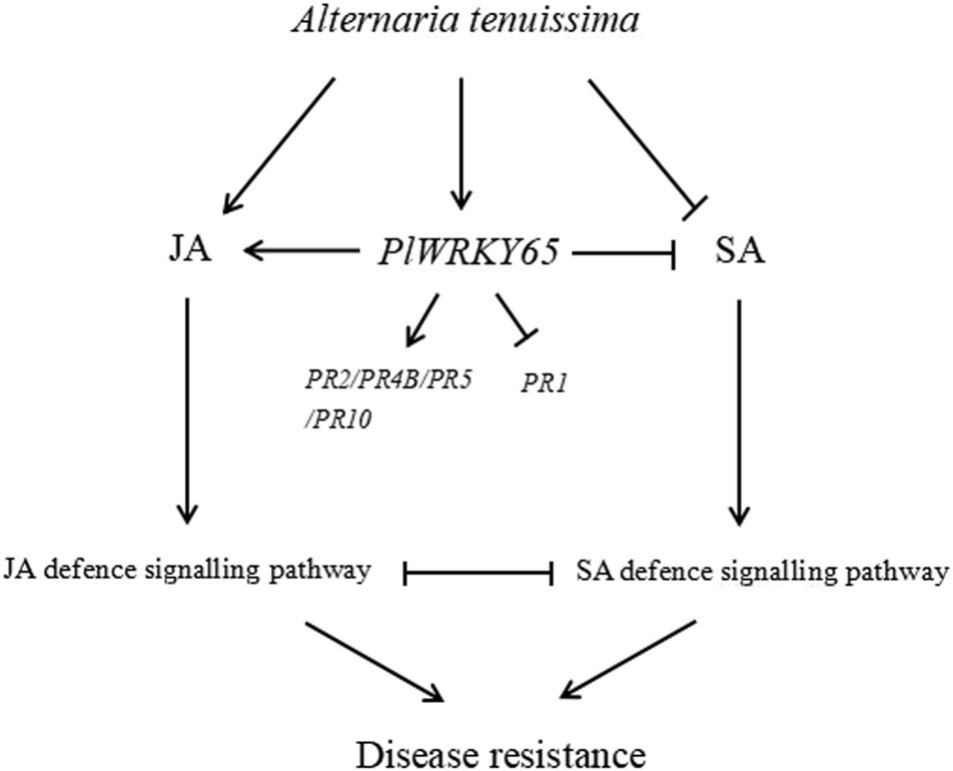
Model diagram of the participation of *PlWRKY65* in the JA regulatory pathway in response to infection with *A. tenuissima*. Solid lines represent the determined regulatory effect, dotted lines represent the regulatory effect of an unknown regulatory mechanism, arrows represent the facilitated effect, and short lines represent inhibition

It can be concluded that *PlWRKY65*, acting as a transcriptional activator responding to pathogen induction, can mediate pathogen resistance by regulating *PlPR* gene expression, which may be partly due to SA-induced and JA-induced resistance. This study broadens our knowledge of the involvement of peony WRKY TFs in pathogen resistance (Fig. 7).

## Materials and methods

### Plant materials and treatments

*P. lactiflora* ‘Da Fugui’ was potted at the experimental forestry station of Shandong Agricultural University, Tai’an, Shandong, China, and plants were selected as experimental materials when they were in the middle stage of leaf development. The wound infection method was adopted for pathogen infection. Strain W23 of *A. tenuissima* preserved in our laboratory was cultured on solid PDA medium for 7 days, and the fungal colony edge was collected as the infection source and transferred to microwounds on leaves punctured with a sterilized insect needle^46^. Leaves were collected at 0, 12, 24, 36, 48, 72, and 96 h after infection. Infection was observed and recorded in 20–30 healthy leaves showing consistent growth for each plant. The incidence grade for peony was calculated according to Li^9^, and the corresponding disease index was calculated as follows: disease index = ∑(number of disease-grade plants × the representative value) × 100%/plant number × the representative value for the most severe disease. All samples were stored at −80 °C after quick freezing with liquid nitrogen. The control group was set up as described above, and each treatment was repeated three times.

*Nicotiana benthamiana* was cultured on an appropriate substrate and placed in a light incubator (25 °C, 120 mol m^−2^ s^−1^, with a light/dark period of 16 h/8 h) for constant-temperature cultivation. When the plants had produced eight true leaves, the subcellular localization test was carried out.

### Total RNA extraction and cDNA synthesis

The total RNA of the plant materials was extracted according to the instructions of the Aidlab EASYspin Rapid RNA Extraction Kit (Aidlab Biotech, Beijing, China). First-strand cDNA synthesis was performed by using the ComWin Biotech Reverse Transcription Kit (ComWin Biotech, Beijing, China).

### Cloning and sequence analysis of the *PlWRKY65* gene

The *PlWRKY65* gene was screened according to the unigene functional annotation of the WRKY genes in the transcriptome data of *P. lactiflora* ‘Da Fugui’; its open-reading frame (ORF) sequence was predicted, and specific primers were designed (Table 1). The total DNA of *P. lactiflora* ‘Da Fugui’ was extracted via the improved CTAB method, and the full-length *PlWRKY65* gene was cloned using total DNA as a template. The NCBI BLAST program (https://blast.ncbi.nlm.nih.gov/Blast.cgi) was used to screen out some amino acid sequences sharing high homology with *P. lactiflora* PlWRKY65, and DNAMAN 5.0 software was then used for multiple-sequence alignment to analyze the structure of the gene domains and conservation. MEGA 5.0 software was used to generate the evolutionary tree of the system for homology analysis.

**Table 1:** Primers used in the tests and their sequences

Primer name	Nucleotide sequence (5′–3′)	Purpose
PlActionF	ACTGCTGAACGGGAAATT	*Actin* primers
PlActionR	ATGGCTGGAACAGGACTT	
PlWRKY65qF	TTTGCCGAAGAGAGAAGCGT	Specific primer for qRT-PCR
PlWRKY65qR	TATACCTCCGTCGCTCCTCC	
PlWRKY65F	ATGGACAGTCTATACAAT	Full-length DNA and cDNA amplification
PlWRKY65R	TCACGAGGTGGTCCCACAC	
PlWRKY65(B)F	CGGGATCCATGGACAGTCTATAC	Vector construction for GFP
PlWRKY65(K)R	GGGGTACCCGAGGTGGTCCCAC	
PlWRKY65(E)F	CGGAATTCAACCACCATGCCACC	Vector construction for VIGS
PlWRKY65(K)R	GGGGTACCCGAGGTGGTCCCACA	
pTRV1F	TTACAGGTTATTTGGGCTAG	Molecular detection of TRV
pTRV1R	CCGGGTTCAATTCCTTATC	
pTRV2F	TTTATGTTCAGGCGGTTCTTGTG	
pTRV2R	CAAACGCCGATCTCAAACAGTC	
PR1F	TACCCAGAGACGGTTCGACT	Primers for pathogenesis-related genes
PR1R	CACACGAGTTGGACCGGTAA	
PR2F	TGGCCAAAGGGGTCTCTAGA	
PR2R	TCCCATTTACGGCAAGCGTA	
PR4BF	ATCCCGCTCAACACTCTTGG	
PR4BR	TCCACAGAAAGCAGTCCACC	
PR5F	CAGTCTTCCCTCAGGCAAGG	
PR5R	GGTTTCACATGCGGGTTTCC	
PR10F	CCGGCAAGGATTTTCAAGGC	
PR10R	TTATCTTGATGGTCCCGGCG	

*Note*: The underlined ‘GGATCC’, ‘GAATTC’, and ‘GGTACC’ nucleotides are the added restriction enzyme recognition sites for BamHI, EcoRI, and KpnI, respectively. Primers for the pTRV1 vector were designed within RNA-dependent RNA polymerase elements (PCR product size of 647 bp in theory), and primers for the pTRV2 vector were designed between MCS elements (PCR product size of 372 bp in theory)

### Subcellular localization

The full-length cDNA of *PlWRKY65* with the termination codon removed was used as a template, and specific primers with restriction sites (BamHI and KpnI) were designed for PCR amplification (Table 1). After enzyme digestion, the obtained product was ligated to the pROKII-GFP vector, which was also double digested, and the fusion expression vector pROKII-*PlWRKY65*-GFP was verified by sequencing. Through *Agrobacterium*-mediated infection, the recombinant vector and the empty vector without *PlWRKY65* were introduced into tobacco leaves. After cultivation for 3 days, fluorescent sites were observed under a confocal laser scanning microscope (Nikon, Tokyo, Japan).

### VIGS in *P. lactiflora*

To specifically silence the *PlWRKY65* gene, we amplified a 402-bp fragment of the gene and cloned it into the pTRV2 vector. The correct recombinant vector was verified by PCR and sequencing.

The pTRV1, pTRV2, and pTRV2-WRKY65 plasmids were transformed into *Agrobacterium tumefaciens* GV3101. A 1 mL aliquot of *A. tumefaciens* GV3101-pTRV1, GV3101-pTRV2, and GV3101-pTRV2-WRKY65 was cultured in 10 mL of YEP liquid medium (including 50 µg/mL Kan and 100 µg/mL Rif) at 28 °C for 24 h at 200 r min^−1^. Then, 10 mL of the bacterial liquid was transferred to 400 mL of YEP liquid medium (including 50 µg/mL Kan, 100 µg/mL Rif, and 200 µM acetosyringone (AS)) followed by culture at 28 °C for 5–6 days at 200 r min^−1^. When the OD_600_ of the bacterial liquid was ~1.5, the cells were centrifuged at 4 °C at 12,000 r/min for 2 min, collected, and resuspended (10 mmol/L MES, 10 mmol/L MgCl_2_, 150 µM AS, and aseptic water as solvent). The OD_600_ of the suspension was adjusted to ~1.5. GV3101-pTRV2 and GV3101-pTRV2-WRKY65 were mixed with GV3101-pTRV1 at a 1:1 ratio, and the mixture was allowed to rest for 3–5 h at room temperature in darkness^47^.

Peonies with strong and consistent growth were selected, and VIGS was carried out via the negative-pressure vacuum filtration method when the underground buds had broken through the soil and were about to produce leaves. The plants were carefully dug out of the pot and placed in a vacuum bucket containing the infection solution described above, ensuring that the plant was completely immersed in the infection solution and that the root system was not destroyed. The treated plants were returned the pots and bagged, then kept in the dark for 24 h. Thereafter, the treated plants and control plants were subjected to normal field management procedures.

### Quantitative real-time PCR (qRT-PCR)

In this experiment, qRT-PCR was used to determine gene expression. The instrument was a Bio-Rad CFX96™ real-time system (Bio-Rad, Hercules, CA, USA), and the qRT-PCR mixture (total volume of 20 μL) contained 10 μL of SYBR® Premix Ex Taq™ (TaKaRa, Inc., Japan), 8 μL of ddH_2_O, 0.5 μL of each primer, and 1 μL of cDNA. The reaction procedure was as follows: 95 °C for 30 s; 40 cycles of 95 °C for 5 s and 60 °C for 30 s; and a dissociation stage of 95 °C for 10 s, 65 °C for 5 s, and 95 °C for 5 s. For each test sample, three biological repeats were performed, and the data were analyzed using the 2^−ΔΔCT^ method^48^. The *PlActin* gene was used as the housekeeping gene to detect the expression levels of other genes.

### Determination of endogenous hormones

The leaves of *P. lactiflora* were sampled at 0, 12, 24, 48, 72, and 96 h after pathogen infection, and the levels of the endogenous hormones JA and SA were determined by high-performance liquid chromatography^49^. The chromatographic conditions were as follows: for JA, a RIGOL L3000 high-performance liquid chromatography instrument (RIGOL, Suzhou, China) was used with a wavelength of 210 nm and a Kromasil C18 reversed-phase chromatographic column (250 mm × 4.6 mm, 5 micron); the flow rate was 0.8 mL/min, the mobile phase was 1% phosphoric acid (aqueous solution):acetonitrile = 45:55 (V/V), and the sample volume was 10 µL. For SA, a Waters 1525 high-performance liquid chromatography instrument (Waters, Shanghai, China) was used with the fluorescence detector set at an excitation wavelength of 294 nm and emission wavelength of 426 nm and a Kromasil C18 reversed-phase chromatographic column (250 mm*4.6 mm, 5 micron); the flow rate was 0.8 mL/min; the mobile phase was 1% acetic acid solution:methanol = 2:3 (V/V); and the sample volume was 10 µL. Three biological repeats were performed for each sample.

### Statistical analysis

At least three biological replicates were included in the data, and all data were analyzed using ANOVA and Student’s *t*-test for the determination of significant differences by using SPSS 24.0 software.
